# Supplementary material for: End Sequence Analysis Toolkit (ESAT) expands the extractable information from single-cell RNA-seq data
Source: Genome Res. 2016 Oct;26(10):1397–410. doi: 10.1101/gr.207902.116 (PMC5052061; doi:10.1101/gr.207902.116)
Supplement: Supplemental Material [file supp_gr.207902.116_Supp_Materials.tar.gz › ESAT-esat_v0.1/doc/html/classumms_1_1core_1_1alignment_1_1_abstract_paired_end_alignment.html]

ESAT: umms.core.alignment.AbstractPairedEndAlignment Class Reference


ESAT


- Main Page
- Related Pages
- Packages
- Classes
- Files

- Class List
- Class Index
- Class Hierarchy
- Class Members

All Classes Namespaces Files Functions Variables Enumerator Pages

- umms
- core
- alignment
- AbstractPairedEndAlignment

Classes |
Public Member Functions |
Static Protected Member Functions |
List of all members

umms.core.alignment.AbstractPairedEndAlignment Class Referenceabstract

Inheritance diagram for umms.core.alignment.AbstractPairedEndAlignment:

[legend]

Collaboration diagram for umms.core.alignment.AbstractPairedEndAlignment:

[legend]

|  |  |
| --- | --- |
| Classes | |
| enum | **TranscriptionRead** |
|  | |

|  |  |
| --- | --- |
| Public Member Functions | |
|  | AbstractPairedEndAlignment (BasicAnnotation b) |
|  | |
| void | refreshAttributeMap () |
|  | |
| String | getReadName () |
|  | |
| String | getChromosome () |
|  | |
| int | getMappingQuality () |
|  | |
| int[] | getIntervalBetweenReads () |
|  | |
| boolean | isPaired () |
|  | |
| boolean | isNegativeStrand () |
|  | |
| boolean | isDuplicate () |
|  | |
| void | setDuplicateFlag (boolean duplicateFlag) |
|  | |
| Alignment | getFirstMate () |
|  | |
| Alignment | getSecondMate () |
|  | |
| int | getFragmentMidpoint (Annotation annot) |
|  | |
| Collection<?extends Window > | getFragment (CoordinateSpace C) |
|  | |
| Strand | getFragmentStrand () |
|  | |
| void | setFragmentStrand (TranscriptionRead transcriptionRead) |
|  | |
| Collection< Integer > | getFragmentSize (CoordinateSpace C) |
|  | |
| int | getFragmentStart () |
|  | |
| int | getFragmentEnd () |
|  | |
| void | setAttribute (String attribute, String value) |
|  | |
| String | getAttribute (String attribute) |
|  | |
| Annotation | getReadAlignmentBlocks (CoordinateSpace C) |
|  | |
| Collection< Alignment > | getReadMates () |
|  | |
| String | toString () |
|  | |
| int | getStart () |
|  | |
| int | getEnd () |
|  | |
| String | getChr () |
|  | |
| int | getAlignmentStart () |
|  | |
| int | getAlignmentEnd () |
|  | |
| boolean | isMapped () |
|  | |
| boolean | isChimera () |
|  | |
| String | getReadSequence () |
|  | |
| double | getWeight () |
|  | |
| boolean | isProperPair () |
|  | |
| void | setProperPairFlag (boolean properPairFlag) |
|  | |
| void | shift (int delta) |
|  | |
| void | moveToCoordinate (int coord) |
|  | |
| final SAMRecord | toSAMRecord () |
|  | |
| Strand | getOrientation () |
|  | |
| Collection< Annotation > | getReadAlignments (CoordinateSpace space) |
|  | |
| boolean | hasIndel () |
|  | |
| int | getFirstFragmentPositionStranded () |
|  | |
| int | getLastFragmentPositionStranded () |
|  | |
| void | setHeader (SAMFileHeader header) |
|  | |
| SAMFileHeader | getHeader () |
|  | |
| Public Member Functions inherited from umms.core.annotation.BasicAnnotation | |
|  | BasicAnnotation () |
|  | |
|  | BasicAnnotation (SAMRecord samRecord) |
|  | |
|  | BasicAnnotation (String ucsc) |
|  | |
|  | BasicAnnotation (String referenceName, int start, int end, Strand orientation, String name) |
|  | |
|  | BasicAnnotation (String referenceName, Strand orientation, String name, Collection<?extends Annotation > blocks) |
|  | |
|  | BasicAnnotation (String referenceName, int start, int end) |
|  | |
|  | BasicAnnotation (String referenceName, int start, int end, Strand orientation) |
|  | |
|  | BasicAnnotation (String referenceName, int start, int end, String orientation) |
|  | |
|  | BasicAnnotation (BasicAnnotation other) |
|  | |
|  | BasicAnnotation (Annotation other) |
|  | |
|  | BasicAnnotation (Collection<?extends Annotation > blocks) |
|  | |
|  | BasicAnnotation (Collection<?extends Annotation > blocks, Strand orientation, String name) |
|  | |
|  | BasicAnnotation (Collection<?extends Annotation > blocks, String name) |
|  | |
|  | BasicAnnotation (String referenceName, CompoundInterval blocks, Strand orientation, String name) |
|  | |
|  | BasicAnnotation (String referenceName, CompoundInterval blocks, Strand orientation) |
|  | |
|  | BasicAnnotation (String referenceName, CompoundInterval blocks) |
|  | |
| Annotation | copy () |
|  | |
| int | getStart () |
|  | |
| int | getEnd () |
|  | |
| int | getMidpoint () |
|  | |
| String | getReferenceName () |
|  | |
| String | getName () |
|  | |
| Strand | getOrientation () |
|  | |
| boolean | isUnoriented () |
|  | |
| List<?extends Annotation > | getBlocks () |
|  | |
| List<?extends Annotation > | getBlocks (boolean oriented) |
|  | |
| Annotation[] | getFlankingBlocks (Annotation spliceJunction) |
|  | |
| int | numBlocks () |
|  | |
| int | length () |
|  | |
| int | getReferenceCoordinateAtPosition (int positionInAnnotation, boolean ignoreOrientation) |
|  | |
| int | getPositionAtReferenceCoordinate (int referenceCoordinate, boolean ignoreOrientation) |
|  | |
| double | getScore () |
|  | |
| String | toString () |
|  | |
| void | setStart (int start) |
|  | |
| void | setEnd (int end) |
|  | |
| void | setOrientation (Strand orientation) |
|  | |
| void | setOrientedStart (int orientedStart) |
|  | |
| void | setOrientedEnd (int orientedEnd) |
|  | |
| void | setReferenceName (String refName) |
|  | |
| void | setName (String n) |
|  | |
| void | setScore (double s) |
|  | |
| void | addBlocks (Annotation block) |
|  | |
| void | addBlocks (Collection<?extends Annotation > blocks) |
|  | |
| void | shift (int delta) |
|  | |
| void | moveToCoordinate (int coordinateInReference) |
|  | |
| boolean | overlaps (Annotation other, int buffer, boolean considerOrientation) |
|  | |
| boolean | overlaps (List<?extends Annotation > otherBlocks, int buffer) |
|  | |
| int | getOverlap (Annotation other) |
|  | |
| boolean | contains (Annotation other) |
|  | |
| Annotation | union (Annotation other) |
|  | |
| Annotation | intersect (Annotation other) |
|  | |
| List< Annotation > | disect (Annotation a) |
|  | |
| List< Annotation > | disect (List<?extends Annotation > disectors) |
|  | |
| Annotation | minus (Annotation other) |
|  | |
| Annotation | minus (Collection<?extends Annotation > others) |
|  | |
| void | stitchTo (Annotation next) |
|  | |
| Annotation | complement () |
|  | |
| Collection<?extends Annotation > | getSpliceConnections () |
|  | |
| Public Member Functions inherited from umms.core.annotation.AbstractAnnotation | |
| int | size () |
|  | |
| int | getSize () |
|  | |
| int | getMidpoint () |
|  | |
| Strand | getStrand () |
|  | |
| boolean | hasOrientation () |
|  | |
| boolean | isNegativeStrand () |
|  | |
| String | getChr () |
|  | |
| int | getOrientedStart () |
|  | |
| int | getOrientedEnd () |
|  | |
| int | getLengthOnReference () |
|  | |
| int | getReferenceCoordinateAtPosition (int positionInAnnotation) |
|  | |
| int | getPositionAtReferenceCoordinate (int referenceCoordinate) |
|  | |
| void | setOrientation (char orientation) |
|  | |
| void | expand (int deltaStart, int deltaEnd) |
|  | |
| Annotation | trim (int deltaStart, int deltaEnd) |
|  | |
| boolean | fullyContains (Annotation other) |
|  | |
| boolean | overlaps (Annotation other) |
|  | |
| boolean | overlaps (Annotation other, int buffer) |
|  | |
| boolean | overlapsStranded (Annotation other) |
|  | |
| boolean | overlaps (Annotation other, boolean considerOrientation) |
|  | |
| boolean | overlaps (Collection<?extends Annotation > others) |
|  | |
| boolean | overlaps (Collection<?extends Annotation > others, int buffer) |
|  | |
| String | toUCSC () |
|  | |
| String | toBED () |
|  | |
| final String | getFullInfoString () |
|  | |
| String | toBED (int r, int g, int b) |
|  | |
| String | toShortBED () |
|  | |
| String | toBEDGraph () |
|  | |
| int | getDistanceTo (Annotation other) |
|  | |
| String | toCigar () |
|  | |
| List< Annotation > | intersect (List<?extends Annotation > others) |
|  | |
| int | compareTo (Annotation other) |
|  | |
| int | compareToAnnotation (Annotation b) |
|  | |
| int | compareToAnnotation (Annotation b, boolean useOrientation) |
|  | |
| boolean | equals (Annotation a) |
|  | |
| boolean | equals (Annotation a, boolean useOrientation) |
|  | |
| int | getSAMStart () |
|  | |
| int | getSAMEnd () |
|  | |
| Public Member Functions inherited from umms.core.alignment.Alignment | |
| Collection<?extends Annotation > | getSpliceConnections () |
|  | |

|  |  |
| --- | --- |
| Static Protected Member Functions | |
| static BasicAnnotation | asAnnotation (SingleEndAlignment firstMate, SingleEndAlignment secondMate, boolean fullFragment) |
|  | |

|  |  |
| --- | --- |
| Additional Inherited Members | |
| Static Public Member Functions inherited from umms.core.annotation.BasicAnnotation | |
| static Annotation | createFromUCSC (String ucsc) |
|  | |
| static BasicAnnotation | fromFullInfoString (String fullInfoString) |
|  | |
| static void | main (String[] args) throws IOException |
|  | |
| Static Public Member Functions inherited from umms.core.annotation.AbstractAnnotation | |
| static Strand | getStrand (String orientation) |
|  | |
| static Strand | getStrand (char orientation) |
|  | |
| Static Public Attributes inherited from umms.core.annotation.AbstractAnnotation | |
| static final int | MAX\_DISTANCE = 1000000000 |
|  | |
| Protected Attributes inherited from umms.core.annotation.BasicAnnotation | |
| CompoundInterval | blocks = new CompoundInterval() |
|  | |

## Detailed Description

Author
:   prussell

## Constructor & Destructor Documentation

|  |  |  |  |  |  |
| --- | --- | --- | --- | --- | --- |
| umms.core.alignment.AbstractPairedEndAlignment.AbstractPairedEndAlignment | ( | BasicAnnotation | *b* | ) |  |

Parameters
:   |  |  |
    | --- | --- |
    | b |  |

## Member Function Documentation

|  |  |  |  |
| --- | --- | --- | --- |
| static BasicAnnotation umms.core.alignment.AbstractPairedEndAlignment.asAnnotation | ( | SingleEndAlignment | *firstMate*, |
|  |  | SingleEndAlignment | *secondMate*, |
|  |  | boolean | *fullFragment* |
|  | ) |  |  |

staticprotected

Get the alignment as an Annotation object

Parameters
:   |  |  |
    | --- | --- |
    | firstMate | First mate |
    | secondMate | Second mate |
    | fullFragment | Whether to get full fragment as one block or separate reads as blocks |

Returns
:   An annotation consisting of the aligned coordinates

Here is the call graph for this function:

Here is the caller graph for this function:

|  |  |  |  |  |
| --- | --- | --- | --- | --- |
| int umms.core.alignment.AbstractPairedEndAlignment.getAlignmentEnd | ( |  | ) |  |

Returns the end position of the alignment

Returns

Implements umms.core.alignment.Alignment.

Here is the call graph for this function:

|  |  |  |  |  |
| --- | --- | --- | --- | --- |
| int umms.core.alignment.AbstractPairedEndAlignment.getAlignmentStart | ( |  | ) |  |

Returns the start position of the alignment

Returns

Implements umms.core.alignment.Alignment.

Here is the call graph for this function:

|  |  |  |  |  |  |
| --- | --- | --- | --- | --- | --- |
| String umms.core.alignment.AbstractPairedEndAlignment.getAttribute | ( | String | *attribute* | ) |  |

Returns the value of the specified attribute

Implements umms.core.alignment.Alignment.

|  |  |  |  |  |
| --- | --- | --- | --- | --- |
| String umms.core.alignment.AbstractPairedEndAlignment.getChr | ( |  | ) |  |

Get chromosome

Returns

Implements umms.core.alignment.Alignment.

Here is the caller graph for this function:

|  |  |  |  |  |
| --- | --- | --- | --- | --- |
| String umms.core.alignment.AbstractPairedEndAlignment.getChromosome | ( |  | ) |  |

Returns the chromosome for this alignment

Returns
:   Reference name

|  |  |  |  |  |
| --- | --- | --- | --- | --- |
| int umms.core.alignment.AbstractPairedEndAlignment.getEnd | ( |  | ) |  |

Get end of the fragment

Returns

Implements umms.core.alignment.Alignment.

|  |  |  |  |  |
| --- | --- | --- | --- | --- |
| int umms.core.alignment.AbstractPairedEndAlignment.getFirstFragmentPositionStranded | ( |  | ) |  |

Get the beginning position of the fragment considering strand

Returns
:   Highest position if negative strand, lowest position otherwise

Implements umms.core.alignment.Alignment.

Here is the call graph for this function:

|  |  |  |  |  |
| --- | --- | --- | --- | --- |
| Alignment umms.core.alignment.AbstractPairedEndAlignment.getFirstMate | ( |  | ) |  |

Get the first read

Returns
:   The first read in pair

Here is the caller graph for this function:

|  |  |  |  |  |  |
| --- | --- | --- | --- | --- | --- |
| Collection<? extends Window> umms.core.alignment.AbstractPairedEndAlignment.getFragment | ( | CoordinateSpace | *C* | ) |  |

Returns an object for the fragment between the read pair in the specified coordinate space

Implements umms.core.alignment.Alignment.

Here is the call graph for this function:

|  |  |  |  |  |
| --- | --- | --- | --- | --- |
| int umms.core.alignment.AbstractPairedEndAlignment.getFragmentEnd | ( |  | ) |  |

Returns the end of the fragment

Implements umms.core.alignment.Alignment.

Here is the call graph for this function:

Here is the caller graph for this function:

|  |  |  |  |  |  |
| --- | --- | --- | --- | --- | --- |
| int umms.core.alignment.AbstractPairedEndAlignment.getFragmentMidpoint | ( | Annotation | *annot* | ) |  |

Get midpoint of fragment with respect to coordinate space

Parameters
:   |  |  |
    | --- | --- |
    | annot | Parent annotation |

Returns
:   Fragment midpoint in coordinate space

Implements umms.core.alignment.Alignment.

|  |  |  |  |  |  |
| --- | --- | --- | --- | --- | --- |
| Collection<Integer> umms.core.alignment.AbstractPairedEndAlignment.getFragmentSize | ( | CoordinateSpace | *C* | ) |  |

Returns the size of the fragment

Implements umms.core.alignment.Alignment.

Here is the call graph for this function:

|  |  |  |  |  |
| --- | --- | --- | --- | --- |
| int umms.core.alignment.AbstractPairedEndAlignment.getFragmentStart | ( |  | ) |  |

Returns the start of the fragment

Implements umms.core.alignment.Alignment.

Here is the call graph for this function:

Here is the caller graph for this function:

|  |  |  |  |  |
| --- | --- | --- | --- | --- |
| Strand umms.core.alignment.AbstractPairedEndAlignment.getFragmentStrand | ( |  | ) |  |

Returns the strand for the fragment, depending on the transcription read

Implements umms.core.alignment.Alignment.

Here is the caller graph for this function:

|  |  |  |  |  |
| --- | --- | --- | --- | --- |
| SAMFileHeader umms.core.alignment.AbstractPairedEndAlignment.getHeader | ( |  | ) |  |

Returns the SAM file header for this alignment

Returns

Implements umms.core.alignment.Alignment.

Here is the call graph for this function:

|  |  |  |  |  |
| --- | --- | --- | --- | --- |
| int [] umms.core.alignment.AbstractPairedEndAlignment.getIntervalBetweenReads | ( |  | ) |  |

Get the coordinates of the first and last position of the interval between the reads

Returns
:   The coordinates of the space between the reads or null if single read or reads overlap

Implements umms.core.alignment.Alignment.

Here is the call graph for this function:

|  |  |  |  |  |
| --- | --- | --- | --- | --- |
| int umms.core.alignment.AbstractPairedEndAlignment.getLastFragmentPositionStranded | ( |  | ) |  |

Get the ending position of the fragment considering strand

Returns
:   Lowest position if negative strand, highest position otherwise

Implements umms.core.alignment.Alignment.

Here is the call graph for this function:

|  |  |  |  |  |
| --- | --- | --- | --- | --- |
| int umms.core.alignment.AbstractPairedEndAlignment.getMappingQuality | ( |  | ) |  |

Returns the sum of the mapping qualities of both mates

Implements umms.core.alignment.Alignment.

Here is the call graph for this function:

|  |  |  |  |  |
| --- | --- | --- | --- | --- |
| Strand umms.core.alignment.AbstractPairedEndAlignment.getOrientation | ( |  | ) |  |

Returns
:   orientation or strand

Implements umms.core.annotation.Annotation.

Here is the call graph for this function:

|  |  |  |  |  |  |
| --- | --- | --- | --- | --- | --- |
| Annotation umms.core.alignment.AbstractPairedEndAlignment.getReadAlignmentBlocks | ( | CoordinateSpace | *C* | ) |  |

This method returns the alignment blocks that the read actually aligns to.

Parameters
:   |  |  |
    | --- | --- |
    | C |  |

Returns

Implements umms.core.alignment.Alignment.

Here is the caller graph for this function:

|  |  |  |  |  |  |
| --- | --- | --- | --- | --- | --- |
| Collection<Annotation> umms.core.alignment.AbstractPairedEndAlignment.getReadAlignments | ( | CoordinateSpace | *space* | ) |  |

This will return the actual read alignments

Parameters
:   |  |  |
    | --- | --- |
    | space | Coordinate Space to return the blocks in |

Implements umms.core.alignment.Alignment.

Here is the call graph for this function:

|  |  |  |  |  |
| --- | --- | --- | --- | --- |
| Collection<Alignment> umms.core.alignment.AbstractPairedEndAlignment.getReadMates | ( |  | ) |  |

This will return the actual read alignment objects NOT BLOCKS

Implements umms.core.alignment.Alignment.

Here is the call graph for this function:

|  |  |  |  |  |
| --- | --- | --- | --- | --- |
| String umms.core.alignment.AbstractPairedEndAlignment.getReadName | ( |  | ) |  |

Returns the name of this read pair.

Implements umms.core.alignment.Alignment.

|  |  |  |  |  |
| --- | --- | --- | --- | --- |
| String umms.core.alignment.AbstractPairedEndAlignment.getReadSequence | ( |  | ) |  |

Implements umms.core.alignment.Alignment.

Here is the call graph for this function:

|  |  |  |  |  |
| --- | --- | --- | --- | --- |
| Alignment umms.core.alignment.AbstractPairedEndAlignment.getSecondMate | ( |  | ) |  |

Get the second read

Returns
:   The second read in pair

Here is the caller graph for this function:

|  |  |  |  |  |
| --- | --- | --- | --- | --- |
| int umms.core.alignment.AbstractPairedEndAlignment.getStart | ( |  | ) |  |

Get start of the fragment

Returns

Implements umms.core.alignment.Alignment.

Here is the caller graph for this function:

|  |  |  |  |  |
| --- | --- | --- | --- | --- |
| double umms.core.alignment.AbstractPairedEndAlignment.getWeight | ( |  | ) |  |

The weight to give this alignment in the scoring. For example, if you want to scale by number of hits, the weight would be 1/NH

Returns

Implements umms.core.alignment.Alignment.

|  |  |  |  |  |
| --- | --- | --- | --- | --- |
| boolean umms.core.alignment.AbstractPairedEndAlignment.hasIndel | ( |  | ) |  |

Returns whether the read contains an insertion or deletion

Returns

Implements umms.core.alignment.Alignment.

|  |  |  |  |  |
| --- | --- | --- | --- | --- |
| boolean umms.core.alignment.AbstractPairedEndAlignment.isChimera | ( |  | ) |  |

For single end, returns false. For paired end, returns true if reads successfully map to different chromosomes.

Returns

Implements umms.core.alignment.Alignment.

Here is the call graph for this function:

|  |  |  |  |  |
| --- | --- | --- | --- | --- |
| boolean umms.core.alignment.AbstractPairedEndAlignment.isDuplicate | ( |  | ) |  |

Returns true is the alignment pair is a duplicate

Implements umms.core.alignment.Alignment.

|  |  |  |  |  |
| --- | --- | --- | --- | --- |
| boolean umms.core.alignment.AbstractPairedEndAlignment.isMapped | ( |  | ) |  |

Returns true if the alignment is mapped

Returns

Implements umms.core.alignment.Alignment.

|  |  |  |  |  |
| --- | --- | --- | --- | --- |
| boolean umms.core.alignment.AbstractPairedEndAlignment.isNegativeStrand | ( |  | ) |  |

For paired end, returns true if the read in direction of transcription is negative stranded

Returns
:   true if the read in direction of transcription is negative stranded

Implements umms.core.alignment.Alignment.

Here is the call graph for this function:

|  |  |  |  |  |
| --- | --- | --- | --- | --- |
| boolean umms.core.alignment.AbstractPairedEndAlignment.isPaired | ( |  | ) |  |

Always returns true for objects of this class.

Implements umms.core.alignment.Alignment.

|  |  |  |  |  |
| --- | --- | --- | --- | --- |
| boolean umms.core.alignment.AbstractPairedEndAlignment.isProperPair | ( |  | ) |  |

Returns whether the alignment represented a proper pair defined by the aligner

Returns

Implements umms.core.alignment.Alignment.

|  |  |  |  |  |  |
| --- | --- | --- | --- | --- | --- |
| void umms.core.alignment.AbstractPairedEndAlignment.moveToCoordinate | ( | int | *coordinateInReference* | ) |  |

Move an annotation, preserving the relationships between its blocks, to a new coordinate.

Parameters
:   |  |  |
    | --- | --- |
    | coordinateInReference |  |

Implements umms.core.annotation.Annotation.

Here is the call graph for this function:

|  |  |  |  |  |
| --- | --- | --- | --- | --- |
| void umms.core.alignment.AbstractPairedEndAlignment.refreshAttributeMap | ( |  | ) |  |

Populate the attribute map with combined attribute values based on the two mates

Here is the caller graph for this function:

|  |  |  |  |
| --- | --- | --- | --- |
| void umms.core.alignment.AbstractPairedEndAlignment.setAttribute | ( | String | *attribute*, |
|  |  | String | *value* |
|  | ) |  |  |

Sets the specified attribute value

Parameters
:   |  |  |
    | --- | --- |
    | attribute |  |
    | value |  |

|  |  |  |  |  |  |
| --- | --- | --- | --- | --- | --- |
| void umms.core.alignment.AbstractPairedEndAlignment.setDuplicateFlag | ( | boolean | *duplicateFlag* | ) |  |

Sets the duplicate flag

Implements umms.core.alignment.Alignment.

|  |  |  |  |  |  |
| --- | --- | --- | --- | --- | --- |
| void umms.core.alignment.AbstractPairedEndAlignment.setFragmentStrand | ( | TranscriptionRead | *transcriptionRead* | ) |  |

Sets the strand for the fragment to the strand of the transcription read

Parameters
:   |  |  |
    | --- | --- |
    | transcriptionRead |  |

Implements umms.core.alignment.Alignment.

Here is the caller graph for this function:

|  |  |  |  |  |  |
| --- | --- | --- | --- | --- | --- |
| void umms.core.alignment.AbstractPairedEndAlignment.setHeader | ( | SAMFileHeader | *header* | ) |  |

Sets the SAM File header for this alignment

Parameters
:   |  |  |
    | --- | --- |
    | header |  |

Implements umms.core.alignment.Alignment.

Here is the call graph for this function:

|  |  |  |  |  |  |
| --- | --- | --- | --- | --- | --- |
| void umms.core.alignment.AbstractPairedEndAlignment.setProperPairFlag | ( | boolean | *properPairFlag* | ) |  |

Set whether the alignment represented a proper pair defined by the aligner

Returns

Implements umms.core.alignment.Alignment.

|  |  |  |  |  |  |
| --- | --- | --- | --- | --- | --- |
| void umms.core.alignment.AbstractPairedEndAlignment.shift | ( | int | *delta* | ) |  |

Shift the positioning of the annotation by a given number of base pairs.

Parameters
:   |  |  |
    | --- | --- |
    | delta | Positive or negative shift to apply to all blocks in the annotation. delta=0  leaves the annotation unchanged. |

Implements umms.core.annotation.Annotation.

Here is the caller graph for this function:

|  |  |  |  |  |
| --- | --- | --- | --- | --- |
| final SAMRecord umms.core.alignment.AbstractPairedEndAlignment.toSAMRecord | ( |  | ) |  |

Implements umms.core.alignment.Alignment.

Here is the call graph for this function:

Here is the caller graph for this function:

|  |  |  |  |  |
| --- | --- | --- | --- | --- |
| String umms.core.alignment.AbstractPairedEndAlignment.toString | ( |  | ) |  |

Here is the call graph for this function:

Here is the caller graph for this function:

---

The documentation for this class was generated from the following file:

- java/umms/core/alignment/AbstractPairedEndAlignment.java


---

Generated on Fri Aug 15 2014 15:11:06 for ESAT by  

 1.8.7
